# Think: Theory for Africa

**DOI:** 10.1371/journal.pcbi.1007049

**Published:** 2019-07-11

**Authors:** Christopher B. Currin, Phumlani N. Khoza, Alexander D. Antrobus, Peter E. Latham, Tim P. Vogels, Joseph V. Raimondo

**Affiliations:** 1 Division of Cell Biology, Department of Human Biology, Neuroscience Institute and Institute of Infectious Disease and Molecular Medicine, Faculty of Health Sciences, University of Cape Town, Cape Town, South Africa; 2 School of Computer Science and Applied Mathematics, University of the Witwatersrand, Johannesburg, South Africa; 3 Gatsby Computational Neuroscience Unit, University College London, London, United Kingdom; 4 Centre for Neural Circuits and Behaviour, Department of Physiology, Anatomy and Genetics, University of Oxford, Oxford, United Kingdom; MIT, UNITED STATES

Understanding the brain is one of the most challenging scientific problems faced by humanity. In addition to offering an improved understanding of who we are, deciphering how our brains operate will provide payoffs for the treatment of mental and neurological disease as well as the creation of intelligent systems. Consequently, developed countries are pouring ever-increasing research funds into brain science. Prominent examples include the BRAIN initiative in the United States and the Human Brain Project in Europe. Although Africa may not yet possess financial and technological resources on this scale, Africans represent a vast and growing human resource poised to make important contributions to neuroscience. As the third yearly International Brain Research Organisation (IBRO)-Simons Computational Neuroscience Imbizo recently wrapped up in South Africa, we call for greater efforts to empower Africans to join the global neuroscience community and argue that the subfield of computational neuroscience is the ideal vehicle to do so.

Computational and theoretical neuroscience, also known as neurotheory, or just “theory” within neurobiology, is a young, rapidly expanding field within neuroscience; its goal is to determine and articulate the general principles by which nervous systems perform their computations. We think it is a good example of how theory, as a subfield, can serve as a catalyst to accelerate the development of science in Africa.

Due to the diversity of methods required for understanding the brain at multiple levels [1], skills are leveraged from a range of fields, such as biology, computer science, electrical engineering, mathematics, physics, and psychology. Beyond just increasing our body of scientific knowledge, the impact of this interdisciplinary field is already significant to the clinic and industry: decoding algorithms are helping restore movement in paralyzed patients [2,3], and machine learning is continually influenced by engagement with neuroscience [4]. A notable aspect of this growing field is its accessibility: computational neuroscience requires only data and individual expertise, independent of geographic location.

There is growing momentum for experimentalists to make their data available for public access and for increased collaboration between groups, a movement lead by flagship projects that curate, systematize, and make freely available large-scale neuronal data [5–8]. A parallel development has been the recent surge in open-access publishing and the establishment of alternative methods for accessing scientific papers outside of well-endowed institutions (e.g., ResearchGate, bioRxiv, and especially important in Africa, SciHub). These dual developments mean that aspiring African scientists in resource-limited settings have increasing access to the latest in both data and theory from across the broad field of neuroscience. From an African perspective, the subspecialty best placed to leverage these developments is computational neuroscience.

The limiting factor for computational neuroscience development in Africa (and anywhere in the world) is quantitative training. Although Africa does not currently contribute a considerable fraction of global neuroscience output (see Fig 1), education in the traditional quantitative disciplines of mathematics, physics, and biology is increasing. African researchers and collaborators are progressively initiating strategies to develop and grow neuroscience expertise through national [9,10], research-domain [11], and education and/or outreach programs [12]. Research-level capacity in quantitative disciplines is being enhanced throughout Africa by initiatives such as the African Institute for Mathematical Science (AIMS), Square Kilometer Array (SKA) [13], the Human Hereditary & Health in Africa (H3Africa) [14], and more neuroscience-focused training organisations such as TReND in Africa and the IBRO. Hallmark features of these initiatives are the integration of local and international expertise as well as sharing of resources. We note also that the confluence of genomics and neuroscience has rich potential for generating unique insights and increasing African capacity in neuroscience [15]. This burgeoning emphasis on quantitative skills development, combined with improved access to data and theory, means that now is the time to put greater effort into facilitating the involvement of Africans in computational neuroscience.

**Fig 1:**
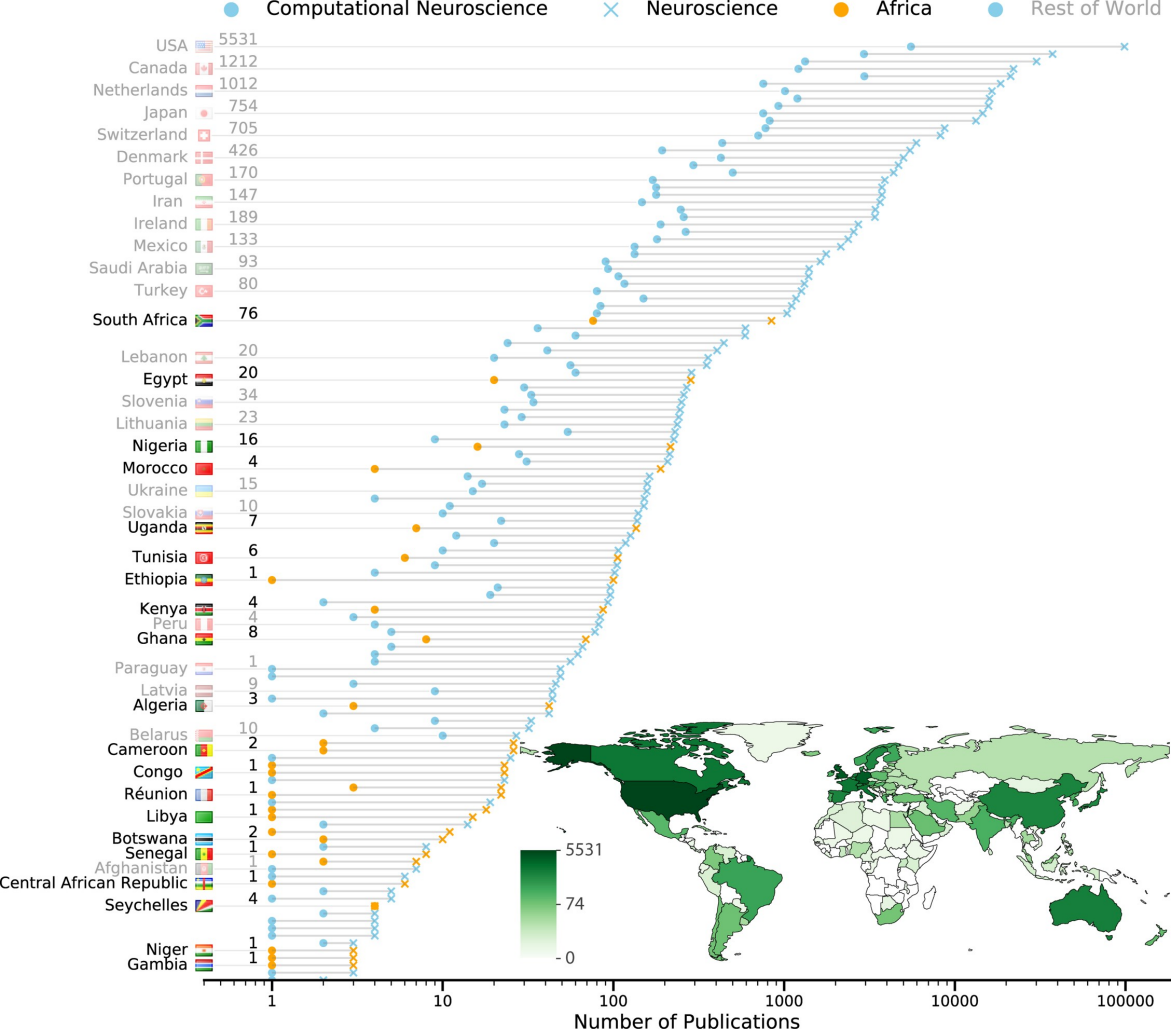
African contributions to neuroscience and computational neuroscience research. Countries are ranked according to the number of neuroscience publications (crosses) generated and indexed on PubMed. The number of computational neuroscience publications produced by each country are indicated by circles. Most contributing African countries (orange, black text) and a subset of global countries (blue, grey text) are labelled on the left. Some African countries (e.g., Ethiopia) have little-to-no computational neuroscience output despite notable neuroscience output. These countries have the potential to leverage existing neuroscience infrastructures for developing computational neuroscience expertise. (Inset) A world map of computational neuroscience publications produced per country, represented as a heat map, highlights the disparity between African nations and the rest of the world. The data were collected through structured searches on PubMed for authors who have been involved in a computational, mathematical, or theoretical neuroscience paper. The code used to generate the figure is available online at https://github.com/ChrisCurrin/comp_neuro_count_countries.

Several promising educational initiatives have recently emerged for Africa. These include annual schools in computational neuroscience (IBRO-Simons Computational Neuroscience Imbizo, isiCNI, almost 100 students trained to date) and machine learning (Deep Learning Indaba, DL Indaba, nearly 1,000 attendees), which both started in 2017. These initiatives bring young, predominantly African scientists together with leading international experts in their field. It is the relationships between students, and particularly between students and faculty, that will support the careers of these fledgling, African computational neuroscientists and machine learning practitioners.

Entering the field of computational neuroscience can be a daunting prospect; there are a proliferation of online courses, numerous textbooks, diverse conferences, and several summer schools. In addition, mailing lists and social media have become an integral component for engaging with current discussions in the field, connecting with authors, and sharing opportunities and information. Although the type of quantitative training background can inform a focus in the field, there are valuable general principles to be gained from the resources in Table 1, especially for those unsure of where to start. Lastly, although research in computational neuroscience is significantly less expensive than experimental neuroscience, there is still some cost involved, mostly for computing resources. Fortunately, many universities in Africa do have high performance computing clusters, and there is the freely available Neuroscience Gateway [16], which offers computational neuroscience-specific computing resources.

**Table 1:** Quickstart guide to computational neuroscience.

Online courses	Computational Neuroscience (Coursera)	Simulation Neuroscience (edX)	Encyclopedia: Computational neuroscience (Scholarpedia)
**Textbooks **	Theoretical Neuroscience: Computational and Mathematical Modeling of Neural Systems by Peter Dayan and Laurence F. Abbott	Information Theory, Inference and Learning Algorithms by David J. C. MacKay	Neuronal Dynamics: From single neurons to networks and models of cognition by Wulfram Gerstner, Werner M. Kistler, Richard Naud, and Liam Paninski
**Schools **	IBRO-Simons Computational Neuroscience Imbizo Cape Town, South Africa	Deep Learning Indaba, Africa, (Location changes annually)	Methods in Computational Neuroscience, Woods Hole, US
**Conferences **	Computational and Systems Neuroscience (COSYNE)	Neural Coding, Computation and Dynamics (NCCD)	Bernstein Conference
**Mailing Lists**	Comp-Neuro	Connectionists	Machine Learning and Data Science Africa

Thus far, the major strides in computational neuroscience development in Africa have come from foreign sources. To retain these initiatives and benefit from these pilot projects, African governments and academic institutions must also be willing to support projects, both financially and logistically. In addition to increasing focus on the field for African scientists, we provide several recommendations for the global computational neuroscience community to enhance the field in Africa:

1. Make funding available for Africans to attend international computational neuroscience conferences, workshops, and summer schools by providing cheaper (or free) registration and travel grants.
2. Support or organise computational neuroscience schools and workshops in Africa.
3. Provide exchange opportunities for students to visit world-class research labs, and, on the other side of the exchange, bring foreign researchers to experience and enhance an African research environment.
4. Make available and accessible teaching material and computational resources.
5. Collaborate with African theoretical and experimental researchers.
6. Incorporate computational neuroscience research groups into African academic institutions.

In the century of the brain, African scientists and educators are poised to make important contributions to global neuroscience research. We believe that theoretical sciences, and specifically computational and theoretical neuroscience are the ideal discipline for the African continent.

## References

[1] Marr D, Poggio T. From Understanding Computation to Understanding Neural Circuitry. Massachusetts Institute of Technology Artificial Intelligence Laboratory 1976 p. 1–22.

[2] Fakhoury M. Neural prostheses for restoring functions lost after spinal cord injury. Neural Regen Res. 2015;10(10):1594–5. 10.4103/1673-5374.165267 26692853 PMC4660749

[3] Yanagisawa T, Hirata M, Saitoh Y, Kishima H, Matsushita K, Goto T, et al Electrocorticographic Control of a Prosthetic Hand in Paralyzed Patients In: Guger C, Allison B, Leuthardt EC, editors. Brain-Computer Interface Research: A State-of-the-Art Summary -2. Berlin, Heidelberg: Springer Berlin Heidelberg; 2014 p. 95–103.

[4] Hassabis D, Kumaran D, Summerfield C, Botvinick M. Neuroscience-Inspired Artificial Intelligence. Neuron. 2017;95(2):245–58. 10.1016/j.neuron.2017.06.011 28728020

[5] Amunts K, Ebell C, Muller J, Telefont M, Knoll A, Lippert T. The Human Brain Project: Creating a European Research Infrastructure to Decode the Human Brain. Neuron. 2016;92(3):574–81. 10.1016/j.neuron.2016.10.046 27809997

[6] Jabalpurwala I. Brain Canada: One Brain One Community. Neuron. 2016;92(3):601–6. 10.1016/j.neuron.2016.10.049 27810001

[7] Martin CL, Chun M. The BRAIN Initiative: Building, Strengthening, and Sustaining. Neuron. 2016;92(3):570–3. 10.1016/j.neuron.2016.10.039 27809996

[8] Sunkin SM, Ng L, Lau C, Dolbeare T, Gilbert TL, Thompson CL, et al Allen Brain Atlas: an integrated spatio-temporal portal for exploring the central nervous system. Nucleic Acids Res. 2013;41(Database issue):D996–1008. 10.1093/nar/gks1042 23193282 PMC3531093

[9] Balogun WG, Cobham AE, Amin A. Neuroscience in Nigeria: the past, the present and the future. Metab Brain Dis. 2018;33(2):359–68. 10.1007/s11011-017-0119-9 28993966

[10] Maina MB, Garba YM, Bukar AM, Ahmad U, Salihu A, Ibrahum H, et al African Neuroscience on the Global Stage: Nigeria as a Model. AfricArXiv. 2018;(7):1–19.

[11] Balogun WG, Cobham AE, Amin A, Seeni A. Advancing Neuroscience Research in Africa: Invertebrate Species to the Rescue. Neuroscience. 2018;374(15 March 2018):323–5. 10.1016/j.neuroscience.2018.01.062 29427653

[12] Baden T, James B, Zimmermann MJY, Bartel P, Grijseels D, Euler T, et al Spikeling: A low-cost hardware implementation of a spiking neuron for neuroscience teaching and outreach. PLoS Biol. 2018;16(10):e2006760 10.1371/journal.pbio.2006760 30365493 PMC6221365

[13] Bhogal N. The role of the Square Kilometre Array in South Africa’s economic development strategy. S Afr J Sci. 2018;114(3):1–7.

[14] Mulder N, Adebamowo CACA, Adebamowo SNSN, Adebayo O, Adeleye O, Alibi M, et al Genomic research data generation, analysis and sharing–challenges in the African setting. Data Sci J. 2017;16(0):1–15.

[15] Karikari TK, Aleksic J. Neurogenomics: An opportunity to integrate neuroscience, genomics and bioinformatics research in Africa. Appl Transl genomics. 2015;5:3–10. 10.1016/j.atg.2015.06.004 PMC4745356 26937352

[16] Sivagnanam S, Majumdar A, Yoshimoto K, Astakhov V, Bandrowski A, Martone M, et al. Introducing the neuroscience gateway. CEUR Workshop Proc. 2013;993. 10.1002/cpe.3283 PMC4624199 26523124

